# Kinsenoside ameliorates intervertebral disc degeneration through the activation of AKT-ERK1/2-Nrf2 signaling pathway

**DOI:** 10.18632/aging.102302

**Published:** 2019-09-23

**Authors:** Yanqiu Wang, Rui Zuo, Ziwen Wang, Liwen Luo, Junlong Wu, Chao Zhang, Minghan Liu, Chunmeng Shi, Yue Zhou

**Affiliations:** 1 Department of Orthopedics, Xinqiao Hospital, Army Medical University (Third Military Medical University), Chongqing 400038, China; 2 Institute of Rocket Force Medicine, State Key Laboratory of Trauma, Burns and Combined Injury, Army Medical University (Third Military Medical University), Chongqing 400038, China

**Keywords:** kinsenoside, intervertebral disc degeneration, apoptosis, senescence, Nrf2, oxidative stress

## Abstract

Intervertebral disc degeneration (IDD) is recognized as the major contributor to low back pain, which results in disability worldwide and heavy burdens on society and economy. Here we present evidence that the lower level of Nrf2 is closely associated with higher grade of IDD. The apoptosis and senescence of nucleus pulposus cells (NPCs) were exacerbated by Nrf2 knockdown, but suppressed by Nrf2 overexpression under oxidative stress. Based on findings that Kinsenoside could exert multiple pharmacological effects, we found that Kinsenoside rescued the NPC viability under oxidative stress and protected against apoptosis, senescence and mitochondrial dysfunction in a Nrf2-dependent way. Further experiments revealed that Kinsenoside activated a signaling pathway of AKT-ERK1/2-Nrf2 in NPCs. Moreover, in vivo study showed that Kinsenoside ameliorated IDD in a puncture-induced model. Together, the present work suggests that Nrf2 is involved in the pathogenesis of IDD and shows the protective effects as well as the underlying mechanism of Kinsenoside on Nrf2 activation in NPCs.

## INTRODUCTION

As an age-related degenerative disease, intervertebral disc degeneration (IDD) has been widely perceived as the main cause of low back pain that occurs in all age groups, bringing about great social and economic burdens [1, 2]. A normal intervertebral disc (IVD) consists of an outer annulus fibrosus (AF) that forms a ring structure to enclose the central nucleus pulposus (NP) and is connected to adjacent vertebral bodies by the cartilaginous endplates. The NP is crucial to maintain biomechanical function of IVD by counteracting and dissipating compressive loads, which depends on the extracellular matrix (ECM) secreted by nucleus pulposus cells (NPCs) [3, 4]. However, the NP changes from a gel-like substance into a fibrous tissue with age, resulting in the structural and functional failure of IVD [5–7]. Although the molecular mechanism of these pathological changes has not been fully understood, the apoptosis and senescence of NPCs are proven to be crucial to the development of IDD [8–10].

At present, increasing studies have demonstrated that reactive oxygen species (ROS) is closely related to the apoptosis and senescence of NPCs, contributing to the initiation and progression of IDD [11–14]. As the main site of intracellular ROS generation, mitochondrion is also adversely influenced by ROS. Mitochondrial dysfunction is regarded as an important factor in NPC apoptosis and senescence, and accelerates disc degeneration [14–16]. Thus, the strategies that aim at antioxidation and maintenance of mitochondrial homeostasis are promising to prevent or retard IDD.

The nuclear factor (erythroid-derived 2)-like 2 (Nrf2) is a basic region-leucine zipper transcription factor that dimerizes with small musculoaponeurotic fibrosarcoma proteins under various stimuli and subsequently activates the target genes to counteract stresses and maintain cellular homeostasis [17, 18]. Hence, numerous studies focus on the roles of Nrf2 in various diseases, including diseases of the lung, liver, kidney, gastrointestinal tract and cardiovascular system [17], considering the abnormal expression and activity of Nrf2 as a key pathomechanism in these diseases. However, little attention has been paid to the relationship between Nrf2 and IDD. Therefore, we hypothesize that Nrf2 may be involved in disc degeneration and can be a therapeutic target for IDD.

Kinsenoside (3-(R)-3-β-D-glucopyranosyloxybutanolide; Kin) is an active monomer extracted from the *Anoectochilus roxburghii*, a traditional Chinese medicine herb that is widespread in tropical regions and known as “the medicine of kings” due to its extensive pharmacological actions. Previous studies showed that Kin exerted beneficial effects in treating gouty arthritis [19], hyperglycemia [20, 21], osteoporosis [22], and autoimmune hepatitis [23]. Notably, other researches indicated that Kin could suppress inflammatory reactions and oxidative stress-induced cell apoptosis [24–26]. Although the pharmacological effects of Kin have been widely explored in diverse diseases, few studies concern the impacts of Kin on Nrf2 modulation and IDD.

In this study, we demonstrated that Kin suppressed apoptosis, senescence and mitochondrial dysfunction in tert-butyl hydroperoxide (TBHP)-treated NPCs by activating the Nrf2, and alleviated IDD in a puncture-induced rat model. Further experiments revealed that Kin promotes the expression and activity of Nrf2 through the activation of AKT and ERK1/2. Importantly, we first established the relationship between Nrf2 and IDD, and elucidated the role of Nrf2 in NPCs under oxidative stress. These findings collectively shed light on the pathogenesis of IDD and provide a novel agonist of Nrf2 to IDD therapy, with evidence for the future clinical translational practice of Kin.

## RESULTS

### Kin suppresses NPC apoptosis and senescence under oxidative stress

**Figure 1:**
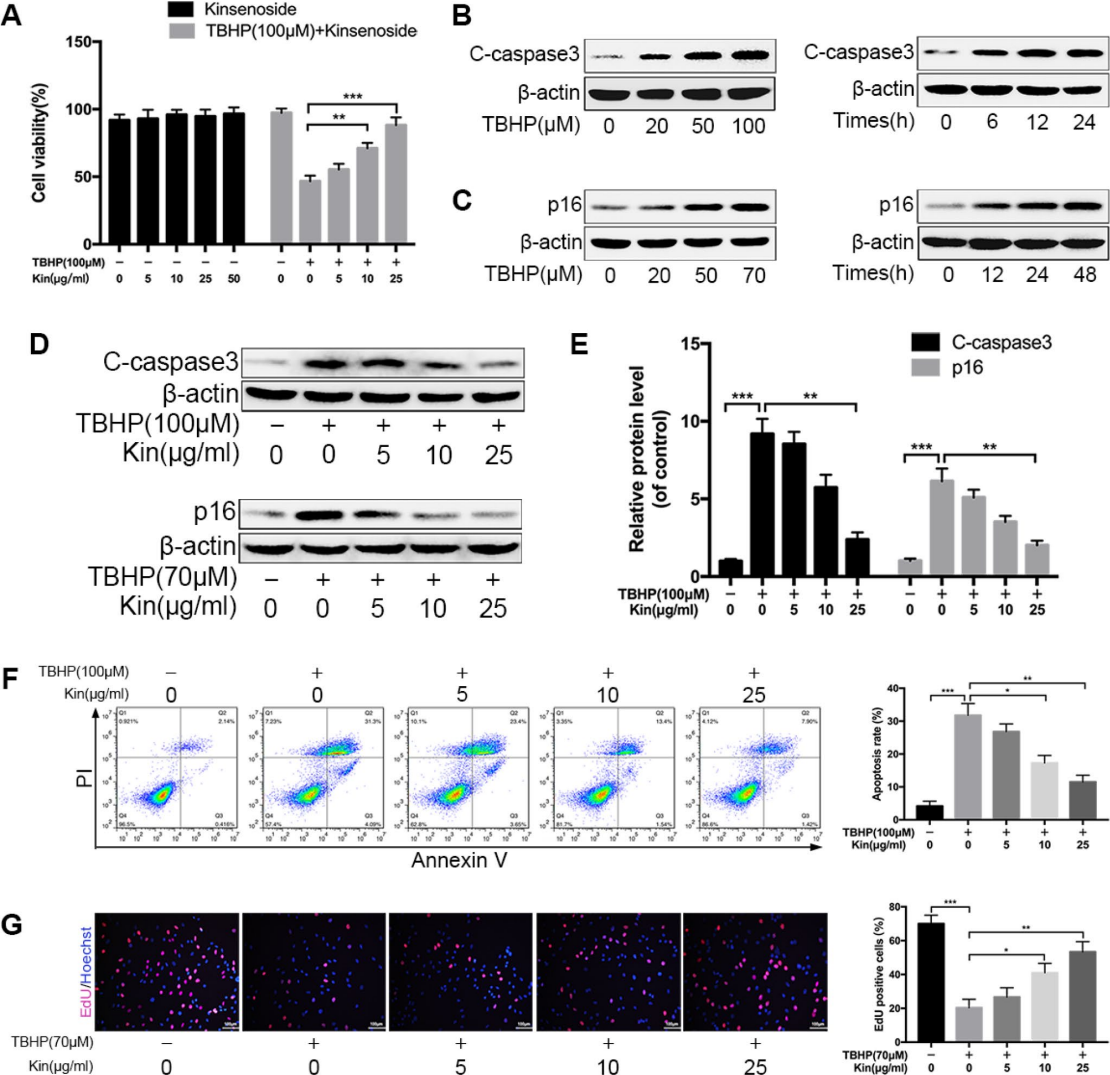
**Kin suppresses NPC apoptosis and senescence under oxidative stress.** (**A**) The NPCs were treated with different concentrations of Kin (0, 5, 10, 25 or 50 μg/ml) alone for 24 h, or Kin (0, 5, 10 or 25 μg/ml) for 2 h before receiving TBHP (100 μM) for 24 h. The cell viability was assessed by CCK-8. (**B**) The western blotting of C-caspase3 in the NPCs treated with different concentrations of TBHP (0, 20, 50 or 100 μM) for 24 h or TBHP (100 μM) for indicated time points (0, 6, 12 or 24 h). (**C**) The western blotting of p16 in the NPCs treated with different concentrations of TBHP (0, 20, 50 or 70 μM) for 48 h or TBHP (70 μM) for indicated time points (0, 12, 24 or 48 h). (**D**, **E**) The western blotting and quantitative protein levels of C-caspase3 and p16 in the NPCs as treated above. (**F**) The apoptosis rate in NPCs, treated with different concentrations of Kin (0, 5, 10 or 25 μg/ml) for 2 h before receiving TBHP (100 μM) for 24h, was analyzed by flow cytometry using Annexin V-APC/PI. (**G**) The cell proliferation was determined by quantification of EdU-positive cells in the NPCs treated with different concentrations of Kin (0, 5, 10 or 25 μg/ml) for 2 h before receiving TBHP (70 μM) for 24 h; scale bar: 100 μm. All data are expressed as mean ± SD of at least three independent experiments.

In this study, we used TBHP to induce oxidative stress, which is a common pathomechanism of NPC apoptosis and senescence [14]. To test the effect of Kin on NPCs viability, we treated NPCs with different concentrations of Kin for 24 h. The CCK-8 results showed that Kin treatment displayed no cytotoxicity in the NPCs and was able to rescue the impaired cell viability under oxidative stress in a dose-dependent manner (Figure 1A). Moreover, the western blotting results showed that Kin could inhibit the protein levels of cleaved-caspase3 (C-caspase3, a protein marker of apoptosis) and p16 (a protein marker of senescence) that were increased by TBHP (Figure 1B–1E). And this anti-apoptotic effect of Kin was further confirmed by the Annexin V-APC/PI staining using flow cytometry (Figure 1F). In addition, the NPC proliferation was determined by EdU staining, which was less positive in senescent cells. As shown in Figure 1G, Kin significantly rescued the capacity of proliferation. Together, these data suggest that Kin could suppress NPC apoptosis and senescence under oxidative stress.

### Kin attenuates TBHP-induced mitochondrial dysfunction in NPCs

**Figure 2:**
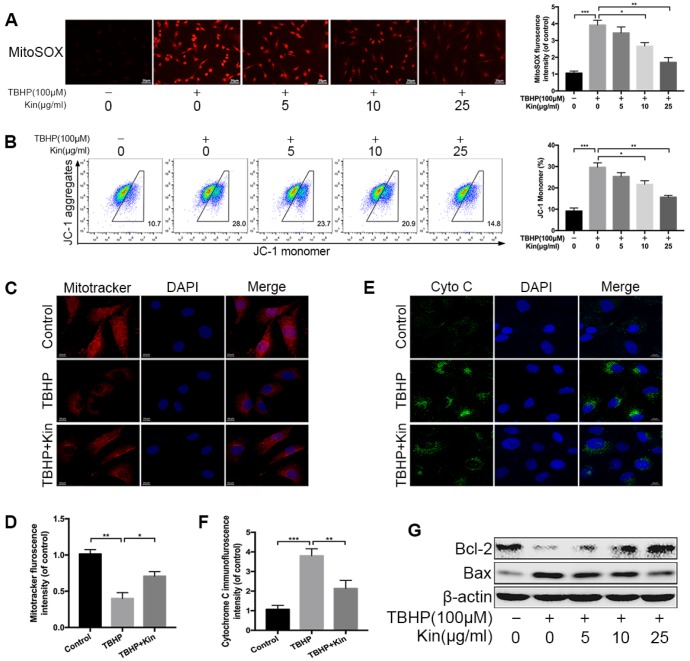
**Kin attenuates TBHP-induced mitochondrial dysfunction in NPCs.** (**A**) The mitochondria-derived ROS in NPCs, treated with different concentrations of Kin (0, 5, 10 or 25 μg/ml) for 2 h before receiving TBHP (100 μM) for 24 h, was detected by MitoSOX staining, and the red fluorescence intensity was quantified; scale bar: 20 μm. (**B**) The mitochondrial membrane potential in NPCs, treated with different concentrations of Kin (0, 5, 10 or 25 μg/ml) for 2 h before receiving TBHP (100 μM) for 24 h, were analyzed by flow cytometry using JC-1 staining. (**C**, **D**) The NPCs were treated with TBHP (100 μM) alone for 24 h, or Kin (25 μg/ml) for 2 h before receiving TBHP (100 μM) for 24 h. The mitochondrial membrane potential was measured by Mitotracker staining and the fluorescence intensity was quantified; scale bar: 10 μm. (**E**, **F**) The immunofluorescence staining of Cyto C in NPCs; scale bar: 10 μm. (**G**) The western blotting of Bcl-2 and Bax in the NPCs treated with different concentrations of Kin (0, 5, 10 or 25 μg/ml) for 2 h before receiving TBHP (100 μM) for 24 h. All data are expressed as mean ± SD of at least three independent experiments.

Mitochondrial dysfunction is involved in the pathogenesis of IDD [14]. When confronting with oxidative stress, mitochondrion is the primary target attacked by ROS, and also produces excessive amounts of ROS due to the damage to enzymes in the electron transport chain [14, 27]. Therefore, we first evaluated the mitochondrial ROS by MitoSOX staining and quantified the fluorescence intensity. The results showed that mitochondrial ROS was markedly increased in TBHP-stimulated NPCs but decreased when the NPCs were pretreated with Kin (Figure 2A). Based on this finding, we wondered if Kin could maintain the mitochondrial membrane potential (MMP), which would be reduced by inner mitochondrial membrane permeabilization. The JC-1 staining and flow cytometry analysis showed that the loss of MMP induced by TBHP was prevented by the administration of Kin in a dose-dependent manner. (Figure 2B). This protective effect on the MMP was further confirmed by the membrane potential-dependent mitochondria staining assay. Pretreatment with Kin significantly increased the fluorescence intensity compared to the NPCs treated with TBHP alone (Figure 2C, 2D). Following the enhanced permeability of mitochondrial membrane, the Cytochrome C (Cyto C), a pro-apoptotic protein that mainly exists in mitochondria, would release into the cytoplasm, contributing to the caspase activation and the apoptotic occurrence. Thus, we determined the cellular localization of Cyto C by immunofluorescence, and we found that Kin inhibited the release of Cyto C under oxidative stress (Figure 2E, 2F). In addition, we also detected the levels of Bcl-2 and Bax, the proteins that activate the outer mitochondrial membrane permeabilization and the mitochondrial apoptotic pathway. The results showed that Kin treatment increased the level of Bcl-2 (an anti-apoptotic protein) but decreased the level of Bax (a pro-apoptotic protein) under oxidative stress (Figure 2G). Collectively, the above data suggest that Kin attenuates TBHP-induced mitochondrial dysfunction in NPCs.

### Kin activates the AKT-ERK1/2-Nrf2 signaling pathway in NPCs

**Figure 3:**
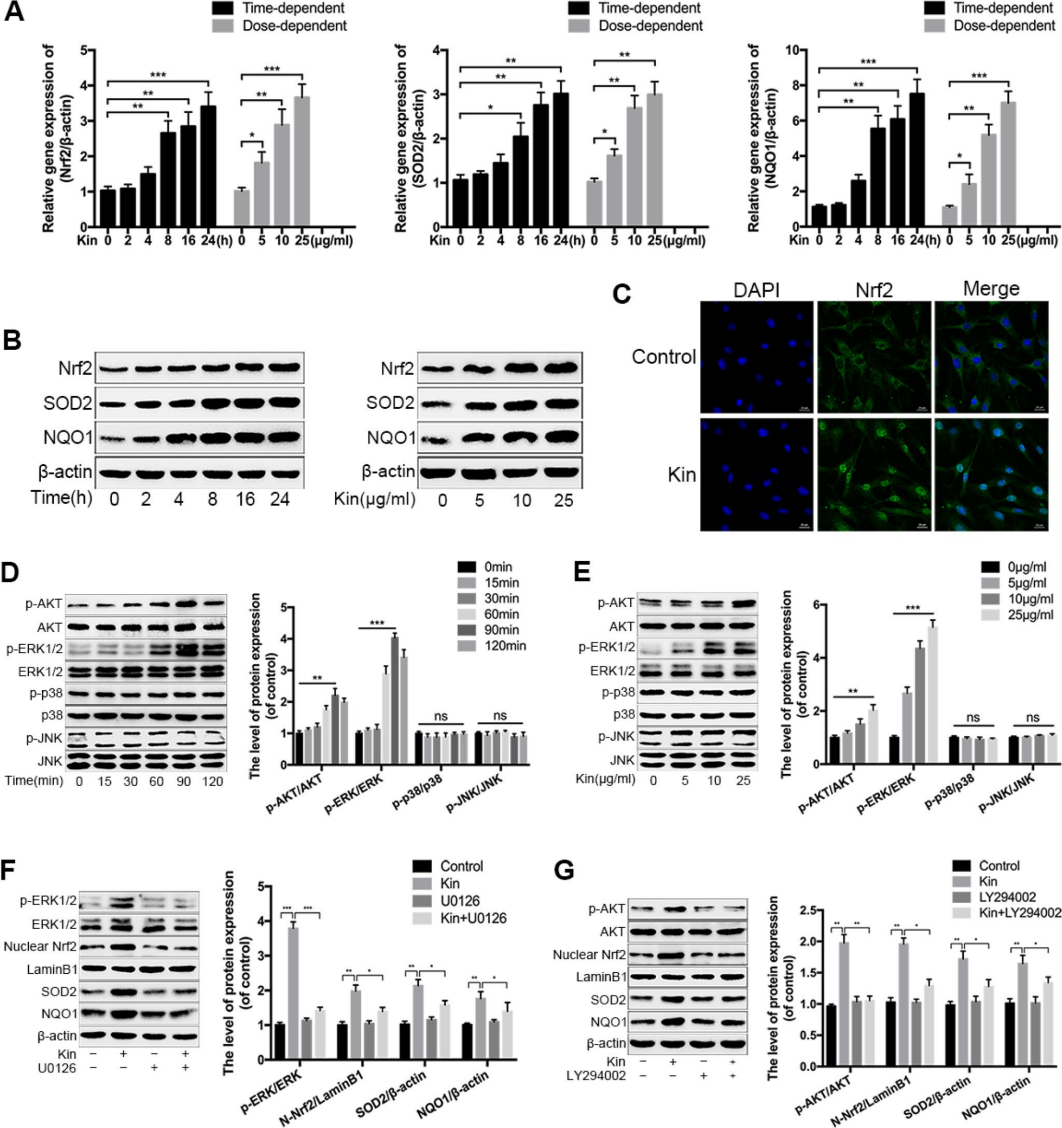
**Kin activates the AKT-ERK1/2-Nrf2 signaling pathway in NPCs.** (**A**) Real-time PCR assay of Nrf2, SOD2 and NQO1 in the NPCs treated with Kin (25 μg/ml) for indicated time points (0, 2, 4, 8, 16 or 24 h) or different concentrations of Kin (0, 5, 10 or 25 μg/ml) for 24 h. (**B**) The western blotting of Nrf2, SOD2 and NQO1 in the NPCs as treated above. (**C**) Nuclear translocation of Nrf2 in the NPCs treated with Kin (25 μg/ml) for 8 h was observed by immunofluorescence staining. (**D**, **E**) The western blotting and quantitative protein levels of p-AKT, AKT, p-ERK1/2, ERK1/2, p-p38, p38, p-JNK and JNK in the NPCs treated with Kin (25 μg/ml) for indicated time points (0, 15, 30, 60, 90 or 120 min) or different concentrations of Kin (0, 5, 10 or 25 μg/ml) for 120 min. (**F**, **G**) The western blotting and quantitative protein levels of p-AKT, AKT, p-ERK1/2, ERK1/2, nuclear Nrf2, SOD2 and NQO1 in the NPCs pretreated with U0126 (10 μM) or LY294002 (10 μM) for 1 h prior to incubation with Kin (25 μg/ml). All data are expressed as mean ± SD of at least three independent experiments.

In view of the antioxidant capacity of Kin, we speculated that Kin could activate the transcription factor Nrf2 and its downstream target genes, endowing NPCs with oxidative stress resistance. To verify this hypothesis, we first measured the expression of Nrf2 and downstream antioxidant enzymes by real-time PCR assay. As shown in Figure 3A, Kin significantly increased the expression of Nrf2 and Nrf2-dependent genes at the mRNA level in a time- and dose-dependent manner. These results were consistent with the western blotting analyses (Figure 3B). In addition, using confocal microscopy, the Nrf2 immunofluorescence in the Kin-treated NPCs exhibited an obvious nuclear translocation of Nrf2 compared to the control group (Figure 3C), indicating the activation of Nrf2. Moreover, to further explored the underlying molecular mechanism of Kin-mediated Nrf2 activation, we also investigated the roles of AKT and MAPK signaling pathways in the regulation of Nrf2 [28]. As shown in Figure 3D, 3E, Kin induced the phosphorylation of upstream kinases, including AKT and ERK1/2, in a time- and dose-dependent manner within 2 h, but without influences on the levels of p-p38 and p-JNK as well as the total protein levels of AKT, ERK1/2, p38 and JNK, suggesting that Kin probably activated Nrf2 through the phosphorylation of AKT and ERK1/2 in NPCs. Therefore, we subsequently applied the U0126 (an ERK1/2 inhibitor) and LY294002 (a PI3K inhibitor) to reduce the activities of these two kinases. The western blotting results showed that pretreatment with LY294002 and U0126 blocked the phosphorylation of AKT and ERK1/2 respectively, and also attenuated the nuclear translocation of Nrf2 in the Kin-treated NPCs (Figure 3F, 3G). Meanwhile, the Kin-induced upregulation of SOD2 and NQO1 was partially suppressed by the pharmacological inhibition of AKT and ERK1/2 respectively (Figure 3F, 3G). Together, the above data indicate that Kin activates Nrf2 through the AKT and ERK1/2 signaling pathways in NPCs.

### Nrf2 knockdown compromises the protective effects of Kin in TBHP-treated NPCs

**Figure 4:**
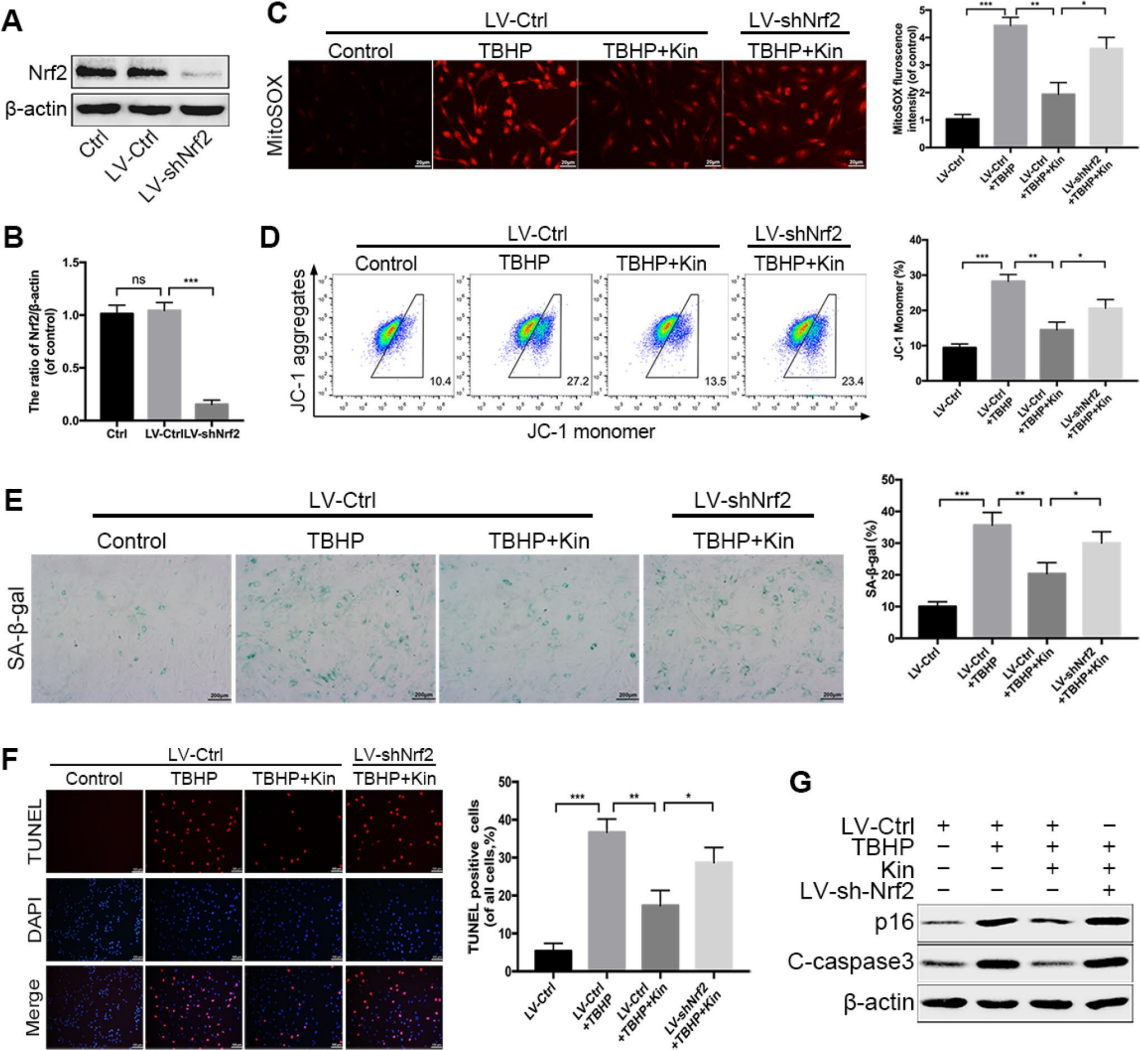
**Nrf2 knockdown compromises the protective effects of Kin in TBHP-treated NPCs.** (**A**, **B**) Transfection efficiency of lentivirus for Nrf2 knockdown was detected by western blotting. (**C**) The mitochondria-derived ROS in treated NPCs was detected by MitoSOX staining, and the red fluorescence intensity was quantified; scale bar: 20 μm. (**D**) The mitochondrial membrane potential in treated NPCs were analyzed by flow cytometry using JC-1 staining. (**E**) The representative images of SA-β-gal staining of NPCs and quantification of positive cells; scale bar: 200 μm. (**F**) The apoptosis of NPCs in each treatment group was measured by the TUNEL assay and TUNEL positive cells were quantified; scale bar: 50 μm. (**G**) The western blotting and quantitative protein levels of p16 and C-caspase3 in the NPCs as treated above. All data are expressed as mean ± SD of at least three independent experiments.

Based on the above findings, we hypothesized that the Kin-mediated cytoprotection in the TBHP-treated NPCs depended on the activation of Nrf2. Thus, we used the lentivirus vector to downregulate the expression of Nrf2 in NPCs before treatments. The transfection efficiency of Nrf2 knockdown was determined by western blotting (Figure 4A, 4B). Then, we measured the mitochondria-derived ROS via MitoSOX staining. The results showed that Nrf2 knockdown diminished the ROS scavenging capacity of Kin under oxidative stress (Figure 4C). In addition, the JC-1 staining analyzed by flow cytometry also indicated that the genetic depletion of Nrf2 could attenuate the protective effect of Kin on the MMP under such condition (Figure 4D). Next, by the SA-β-gal staining assay, we found that the number of senescent NPCs was increased by TBHP, while reduced by Kin via Nrf2 activation (Figure 4E). Meanwhile, the apoptosis evaluation using TUNEL assay coincided with our observations in the senescence assessment (Figure 4F). These results were further confirmed by the changes in protein levels of p16 and C-caspase3 (Figure 4G). Together, the above data suggest that the beneficial effects of Kin on mitochondrial dysfunction, senescence and apoptosis are closely associated with Nrf2 activation in NPCs.

### Nrf2 protects against apoptosis and senescence under oxidative stress in NPCs

**Figure 5:**
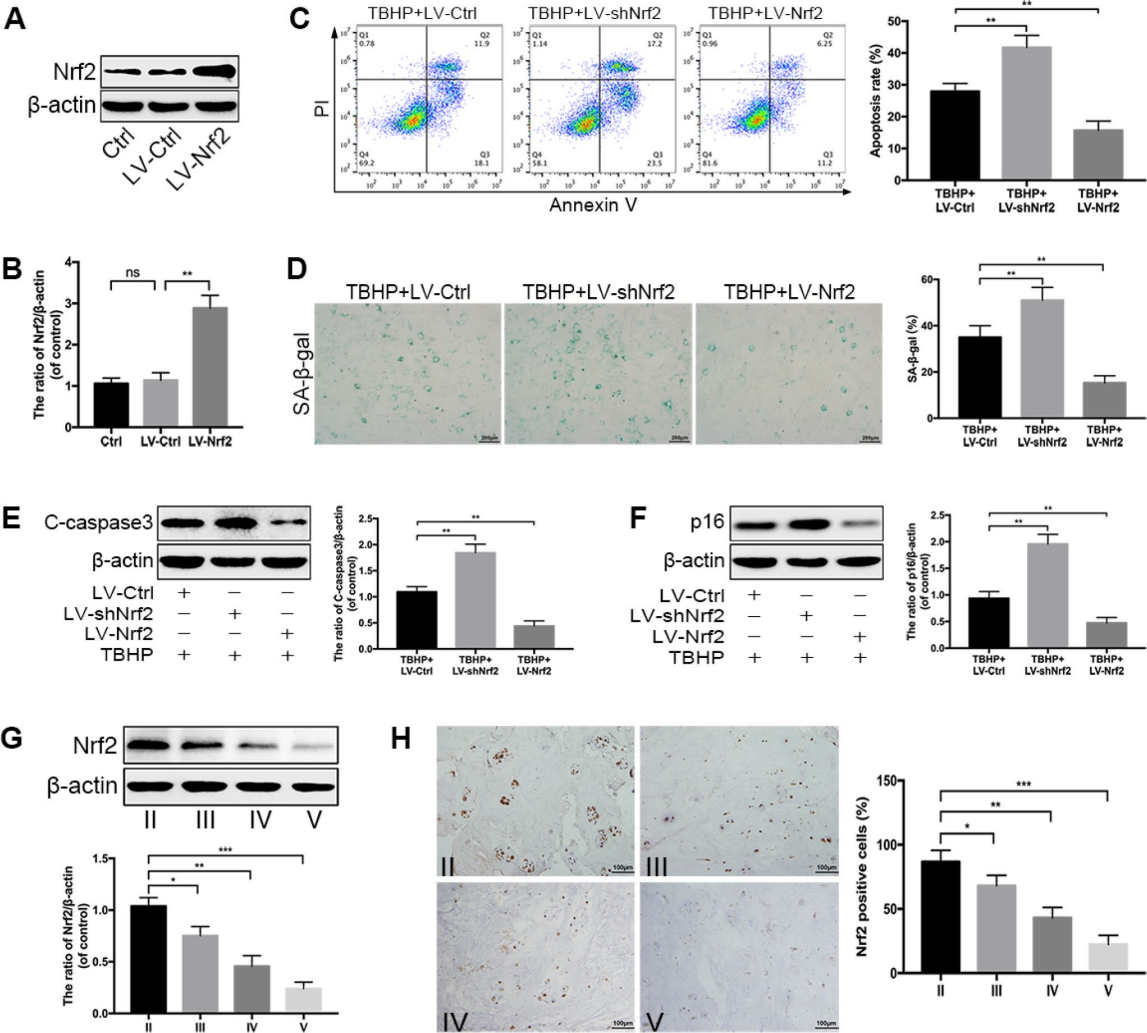
**Nrf2 protects against apoptosis and senescence under oxidative stress in NPCs.** (**A**, **B**) Transfection efficiency of lentivirus for Nrf2 overexpression was detected by western blotting. (**C**) The Annexin V-APC/PI staining analyzed by flow cytometry showed that Nrf2 overexpression reduced the apoptosis rate in TBHP-treated NPCs, which was increased by Nrf2 knockdown. (**D**) The representative images of SA-β-gal staining of NPCs as treated above and quantification of positive cells; scale bar: 200 μm. (**E**, **F**) The western blotting and quantitative protein levels of p16 and C-caspase3 in the NPCs of each treatment group described above. (**G**) The western blotting and quantitative protein levels of Nrf2 in each human NP tissue group (n = 12, 3 for each grade). (**H**) Immunohistochemical staining showed that the numbers of Nrf2-positive cells were decreased with IDD; scale bar: 100 μm. All data are expressed as mean ± SD of at least three independent experiments.

To further clarify the roles of Nrf2 in resisting oxidative stress, we detected the changes in cellular responses induced by TBHP when the expression of Nrf2 was downregulated (LV-shNrf2) or overexpressed (LV-Nrf2) via transfection with lentivirus. The transfection efficiency of Nrf2 overexpression was determined by western blotting (Figure 5A, 5B). Firstly, we quantified the apoptotic cells in treated-NPCs using Annexin-V/PI staining. The results showed that Nrf2 knockdown could increase the apoptosis rate in TBHP-treated NPCs, while LV-Nrf2 transfection could significantly alleviate NPC apoptosis (Figure 5C). The results were also confirmed by the level of C-caspase3 (Figure 5E). In addition, the SA-β-gal staining assay demonstrated that senescence of NPCs could be worsened by genetic ablation of Nrf2, but suppressed by Nrf2 overexpression under oxidative stress (Figure 5D). This finding was correspondingly verified by the western blotting of p16 (Figure 5F). Nrf2 is believed to maintain cellular homeostasis under stress, and its dysregulation has been considered as a key pathomechanism in chronic diseases [17]. However, the relationship between Nrf2 and IDD has not been investigated. Therefore, we collected the human NP tissues from patients with different grades of IDD. The protein level of Nrf2 in human NP samples was measured by western blotting and immunohistochemical staining. As displayed in Figure 5G, 5H, the level of Nrf2 was markedly decreased with the IDD grade. Therefore, the above data suggest that Nrf2 protects against apoptosis and senescence under oxidative stress in NPCs and the decreased Nrf2 protein level negatively correlates with the IDD grade.

### Kin ameliorates IDD in a rat model

**Figure 6:**
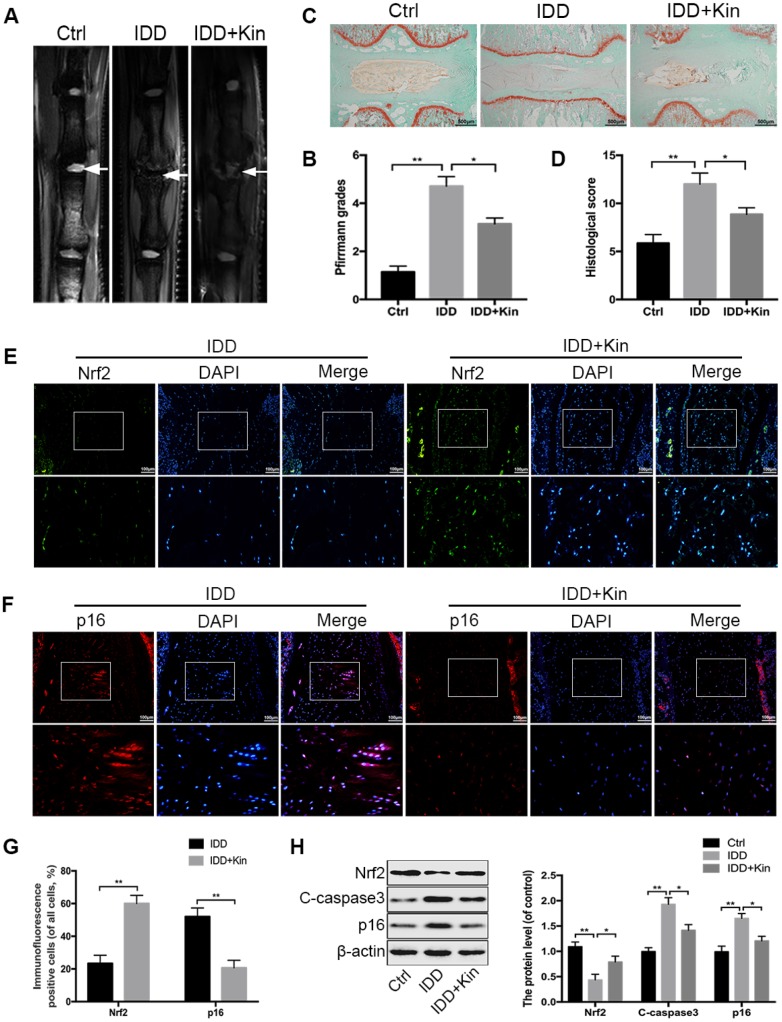
**Kin ameliorates IDD in a rat model.** (**A**, **B**) The extent of IDD in the punctured discs in each group was evaluated by T2-weighted MRI and Pfirrmann grading at four weeks (white arrows). (**C**, **D**) The sections of each disc were stained with Safranin O-fast green and evaluated by the histological score based on the morphology of discs; scale bar: 500 μm. (**E**, **F**) The representative images of immunofluorescence staining of Nrf2 and p16 in the rat disc samples; scale bar: 100 μm. (**G**) The quantification of positive cells in the immunofluorescence staining of Nrf2 and p16. (**H**). The western blotting and quantitative protein levels of Nrf2, C-caspase3 and p16 in the NP samples of rats. All data are expressed as mean ± SD of seven rats per group.

According to the results obtained from in vitro experiments, we wondered whether Kin could be a therapeutic agent for the treatment of IDD in vivo. Then we constructed the IDD model by puncturing caudal discs of rats. The MRI examination was first applied to assess the IDD grade at 4 weeks after surgery. As shown in Figure 6A, there was an obvious loss of T2-weighted signal in punctured discs of the IDD group, which represented the disc degeneration, while the administration of Kin could partly improve the MRI signal intensity. Similarly, the Pfirrmann grades, which also indicate the IDD extent based on the magnetic resonance images, were significantly lower in the Kin-treated group than those in the IDD group (Figure 6B). In addition, the IVD specimens collected from IDD models were subjected to Safranin O/fast green staining and evaluated by the histological score based on the morphology of discs. As seen in Figure 6C, the normal IVD contained a well-organized AF layer and an oval-shaped NP that was rich in proteoglycan and glycosaminoglycan. However, after the surgery, degenerative changes occurred in the IVD, which was characterized by the collapsed disc height, loss of matrix, disorganized AF layer, disappeared boundary between AF and NP, as well as hypocellular fibrocartilaginous tissue found in the NP area (Figure 6C). However, to a certain extent, these pathological features were alleviated by Kin administration, as demonstrated by the better-preserved structure of IVD in the Kin-treated group (Figure 6C). Meanwhile, the histological score was significantly lower in the IDD+Kin group than that in the IDD group, further confirming that Kin could ameliorate the puncture-induced IDD (Figure 6D). Subsequently, we measured the expression of Nrf2 and p16 in the rat NP by immunohistofluorescence and corresponding quantification. The results revealed that Kin administration could induce the upregulation of Nrf2 and the downregulation of p16, comparing to the IDD group (Figure 6E–6G). Moreover, western blotting results showed that Kin treatment increased the level of Nrf2 and decreased the levels of C-caspase3 and p16 (Figure 6H), which further supported our findings in vitro. Taken together, the above data suggest that Kin promotes the expression of Nrf2 and ameliorates IDD in vivo.

## DISCUSSION

IDD is acknowledged as a multifactorial disorder that involves complex signal networks and diverse effector molecules [29, 30]. Thus, it is a priority to understand the pathomechanism of IDD, which may facilitate the drug development and clinical treatment strategies. Recently, increasing studies have demonstrated that the accumulation of oxidation products in NP tissues forms a microenvironment of oxidative stress and contributes to the progression of IDD [31–33]. This evidence suggests that oxidative stress is a crucial mediator of IDD pathogenesis and alleviating oxidative stress is conducive to preventing or retarding IDD [11, 13]. Therefore, we propose a strategy for the treatment of IDD by enhancing the antioxidant capacity of cells.

Because of the multiple pharmacological effects of Kin reported by other research groups, including anti-oxidation, anti-inflammation, anti-apoptosis and so on [19, 25, 26, 34], we hypothesized that Kin could protect NPCs against apoptosis and senescence under oxidative stress. And this was verified by our subsequent experiments. It is well-known that mitochondrial dysfunction is an important characteristic of cellular apoptosis and senescence [35, 36], and serves as a key factor in numerous diseases such as tumors, osteoarthritis and degenerative diseases [37–39]. According to the previous reports, mitochondrial dysfunction is also implicated in the development of IDD [14–16]. Therefore, we wondered whether Kin could attenuate mitochondrial dysfunction in NPCs under oxidative stress. In this study, we observed that Kin could significantly reduce the mitochondrial ROS, which was proven to be necessary for the stress-induced senescence [40]. Moreover, mitochondrial dysfunction can activate the intrinsic pathway of apoptosis that involves the loss of MMP, the imbalance between pro- and anti-apoptotic proteins in the Bcl-2 family, the release of pro-apoptotic molecules such as Cyto C and the downstream activation of caspase family [41]. Therefore, we further investigated the impacts of Kin on the MMP, the release of mitochondrial Cyto C and the levels of Bcl-2 and Bax under oxidative stress. However, multiple mitochondrial factors, besides mitochondrial ROS, such as mitochondrial dynamics, metabolism, respiratory chain, bioenergetic balance, and calcium homeostasis, can induce cellular apoptosis and senescence [35, 41]. Elucidating the detailed mechanisms about their crosstalk may enrich our understanding of mitochondrial diverse yet interconnected roles in determining cell fate, and shed light on the pathogenesis of IDD.

Since Kin could significantly inhibit the deleterious effects induced by oxidative stress, we hypothesized that Kin activated cytoprotective signaling pathways that enhanced the antioxidant capacity of NPCs. As a master regulator of the cellular antioxidant response, Nrf2 controls the expression of ARE-containing genes that counteract the oxidative stress and maintain redox homeostasis [42]. Under unstressed conditions, Nrf2 is a protein of low abundance due to the proteasomal degradation mediated by its principal repressor, the E3 ligase adaptor Kelch-like ECH-associated protein 1 (Keap1) [17]. Intriguingly, we observed that Kin could promote the expression of Nrf2 in a time- and dose-dependent manner and facilitate the nuclear translocation of Nrf2, increasing the transcription of Nrf2 downstream effectors including SOD2 and NQO1. In addition, by investigation of several upstream signaling pathways, we found that Kin activated Nrf2 via the AKT and ERK1/2 instead of p38 and JNK pathways. To further demonstrate that Nrf2 activation mediates the protective effects of Kin in NPCs under oxidative stress, we conducted lentivirus transfection to downregulate the expression of Nrf2 before treatments. Interestingly, Nrf2 knockdown markedly abolished the anti-mitochondrial dysfunction, anti-apoptosis and anti-senescence effects of Kin in TBHP-treated NPCs, which suggested that the cytoprotection of Kin was dependent on the Nrf2 activation.

It is recognized that Nrf2 activation protects against a myriad of diseases that are characterized by inflammation and oxidative stress, including autoimmune and metabolic disorders; neurodegeneration; chronic diseases of the lung and liver [17]. However, we observed that the expression level of Nrf2 was significantly decreased with the development of IDD, suggesting that Nrf2 might be closely bound up with IDD. To further confirm the roles of Nrf2 under oxidative stress, we demonstrated that Nrf2 knockdown aggravated apoptosis, senescence and mitochondrial dysfunction induced by TBHP, while Nrf2 overexpression could partially attenuate these pathological changes in NPCs. Similarly, previous studies have reported that Nrf2 expression declines with aging, while its upregulation using pharmacological or genetic methods improves cell function and increases lifespan [43, 44]. Therefore, we wondered whether Kin could ameliorate the progression of IDD by targeting Nrf2. Using puncture-induced IDD models in rats, we showed that Kin significantly suppressed apoptosis and senescence, promoted Nrf2 expression, and alleviated IDD compared to the group without Kin administration. However, there are several limitations in our research. Firstly, when we analyzed the expression of Nrf2 in human samples, we only considered the impacts of degenerative grade, but neglected the influence of other factors such as age and gender. Furthermore, we did not use transgenic animals, of which the Nrf2 is specifically deleted in IVD, to study the roles of Nrf2 during aging and degeneration. Finally, because our animal model cannot fully reflect the human IDD pathologies, only in the future clinical trials can we accurately testify the therapeutic efficacy of Kin.

**Figure 7:**
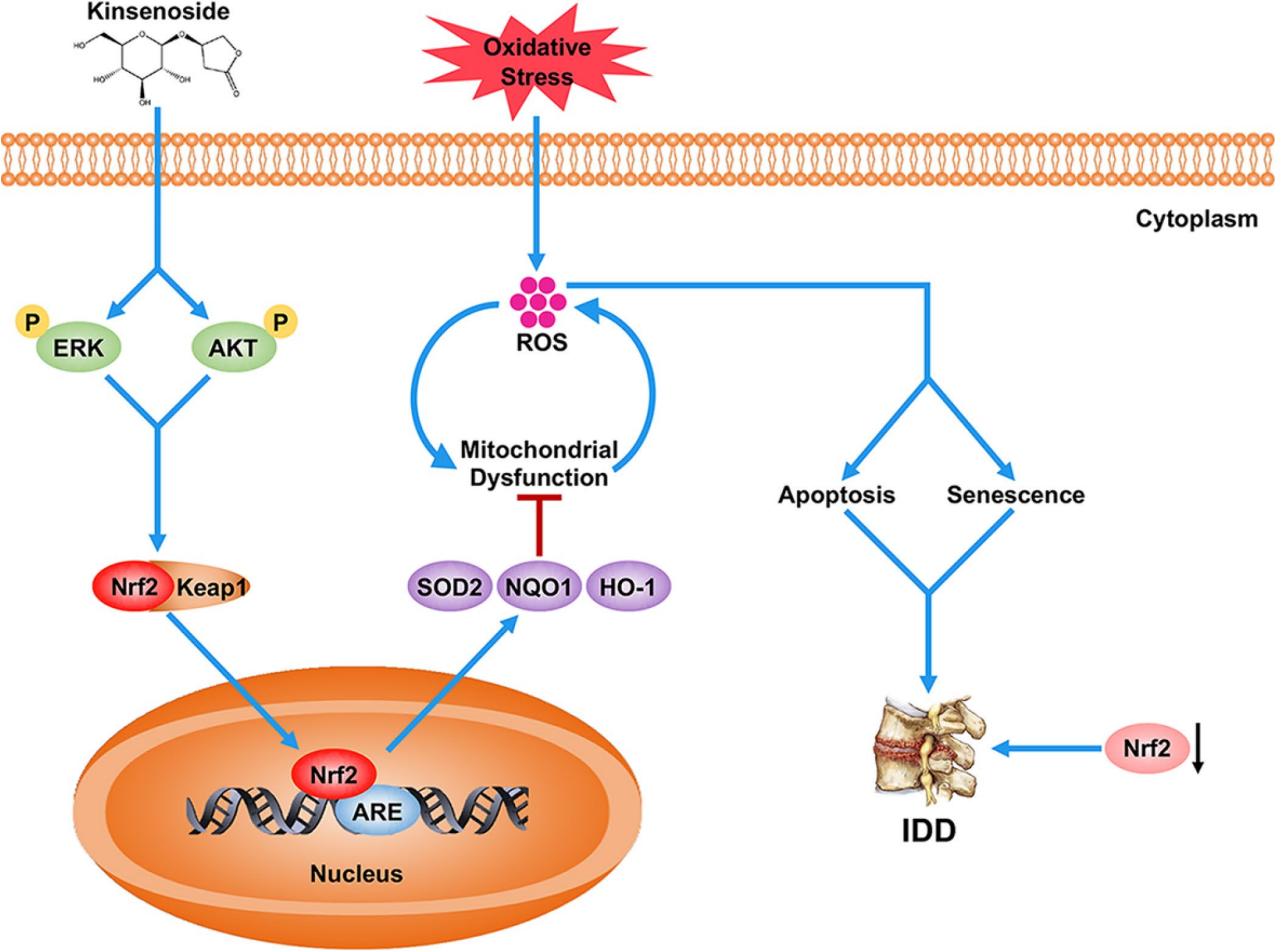
**Schematic representation illustrating the relationship between Nrf2 and IDD as well as the protective effects of Kin on IDD.** Briefly, the decreased Nrf2 expression compromises the antioxidant ability of NPCs, which further contributes to the excessive apoptosis and senescence of NPCs under oxidative stress, and eventually the disc degeneration. However, Kin is able to activate the AKT-ERK1/2-Nrf2 signaling pathway in NPCs, alleviating mitochondrial dysfunction, apoptosis and senescence in vitro and ameliorating IDD in vivo.

In conclusion, the present work demonstrated that Kin enhanced the antioxidant capacity of NPCs via a signaling pathway of AKT-ERK1/2-Nrf2 and ameliorate disc degeneration in both in vitro and in vivo experiments (Figure 7). Moreover, in clinical samples, we also demonstrated that the Nrf2 expression was negatively associated with IDD. These findings provide new insights into the pathogenesis of IDD as well as a novel agonist of Nrf2, suggesting that Nrf2 may serve as a promising target for IDD therapy.

## MATERIALS AND METHODS

### Ethics statement

All the NP tissues used in this study were acquired from lumbar spine surgery patients admitted to the Xinqiao Hospital of Third Military Medical University. The collection of human NP samples and related procedures were approved by the Ethical Committee of the Xinqiao Hospital. The animal experiments in our study were conducted in accordance with the Helsinki Declaration and approved by the Laboratory Animal Welfare and Ethics Committee of Third Military Medical University.

### Reagents and antibodies

The cell culture reagents were purchased from HyClone Laboratories (Logan, UT, USA). Kin (CAS: 151870-74-5) was obtained from Shifeng Biological Technology Company (Shanghai, China). The 4′, 6-diamidino- 2-phenylindole (DAPI), U0126, LY294002 and horseradish peroxidase conjugated secondary antibodies were acquired from Beyotime (Shanghai, China). Antibodies against cleaved-caspase3, AKT, ERK1/2, p38, JNK, p-AKT, p-ERK1/2, p-p38 and p-JNK were purchased from Cell Signaling Technology. Antibodies against β-actin, Bax, Cytochrome c, SOD2, NQO1 and Nrf2 were the products of Proteintech. Antibodies against Collagen I and Collagen II were purchased from Abcam. Antibody against Bcl-2 was provided by Santa Cruz Biotechnology. TBHP, the type II collagenases, p16 antibody and other reagents were obtained from Sigma-Aldrich unless noted otherwise.

### Rat NPC isolation and culture

The translucent and gelatinous NP tissues were isolated from the tails of Sprague-Dawley rats (either sex, 100–150 g) under aseptic conditions and subsequently digested in DMEM/F12 medium containing 0.2% type II collagenase for 4 h at 37 °C. Then, the digested tissues were washed with phosphate-buffered saline (PBS), transferred as explants to DMEM/F12 medium with 10% fetal bovine serum (FBS) and 1% streptomycin/penicillin, and then cultured at 37 °C in an incubator containing 5% CO_2_. When confluent, the primary NPCs were trypsinized with 0.25% trypsin-EDTA and replanted into 10-cm culture dishes at the appropriate density. The culture medium was changed twice a week. The first three passages of cells that were cultured in a monolayer were used for all experiments. To induce cellular senescence, we treated NPCs with relatively lower concentration of TBHP (70 μM) for 48 h. While NPC apoptosis was induced by a higher concentration of TBHP (100 μM) for a shorter time (24 h).

### NPC viability and proliferation assay

The NPC viability was assessed with the cell counting kit-8 (CCK-8; Dojindo Co., Kumamoto, Japan) according to the manufacturer’s protocol. NPCs were planted in 96-well plates and incubated in DMEM/F12 with 10% FBS for 24 h at 37 °C. Next, the cells were treated with different doses of Kin for 24h. To test the effect of Kin on the NPC viability under oxidative stress, the cells were pretreated with different doses of Kin for 2h before TBHP (100 μM) addition for 24h. After treatments, the cells were washed with PBS, and then 10 μl of CCK-8 solution was added to 90 μl of complete medium in each well and continuously incubated for additional 2 h at 37 °C. The absorbance of the wells was measured at 450 nm using a microplate reader (Thermo Scientific, MA, USA). The NPC proliferation was evaluated by a EdU Cell Proliferation Kit (Beyotime). Briefly, the cells were pretreated with different doses of Kin for 2h before TBHP (70 μM) addition for 24h, and then stained with EdU. The images were obtained by a fluorescence microscope (Olympus Inc., Tokyo, Japan).

### Western blotting

The total protein of NP cells was extracted using RIPA buffer (Beyotime) with a cocktail of protease and phosphatase inhibitors (Thermo Scientific, IL, USA). For the nuclear fractions, the proteins were isolated using a nuclear extraction kit (Beyotime) according to the manufacturer’s instructions. Protein concentration was determined by an Enhanced BCA Protein assay kit (Beyotime). Equivalent amounts of lysate protein (30 μg) were separated on sodium dodecyl sulphate-polyacrylamide gel electrophoresis (SDS-PAGE) and transferred to polyvinylidene difluoride membranes (Millipore, MO, USA) followed by blocking with Western Blocking Buffer (Beyotime). Next, the bands were incubated with primary antibodies specific to Nrf2 (1:1000), cleaved-caspase3 (1:1000), p16 (1:1000), Bcl-2 (1:1000), Bax (1:1000), SOD2 (1:1000), NQO1 (1:1000), p-AKT (1:1000), AKT (1:1000), p-ERK1/2 (1:1000), ERK1/2 (1:1000), p-p38 (1:1000), p38 (1:1000), p-JNK (1:1000), JNK (1:1000), LaminB1 (1:1000) and β-actin (1:1000) overnight at 4 °C, before incubation with respective secondary antibodies for 2 h at room temperature. After that, the bands were washed three times with TBST (Tris Buffered Saline with Tween 20) for 10 min and subsequently visualized by ECL Plus Reagent (Beyotime) using Image Lab 3.0 software (Bio-Rad, CA, USA). Finally, the intensity of these bands was analyzed by ImageJ software (Bethesda, MD, USA), and the data were normalized to β-actin expression.

### Apoptosis assay by Annexin V/PI

Apoptosis rate was assessed by flow cytometry with the Annexin V-APC/PI apoptosis detection kit (BD Biosciences, CA, USA). Briefly, NPCs were pretreated with different doses of Kin for 2h before TBHP (100 μM) addition for 24h. Then the treated NPCs were harvested, washed twice with PBS, and resuspended in 100 μl binding buffer mixed with 5 μl of Annexin V-APC and 5 μl of PI. After incubation at room temperature in the dark for 15 min, the cells were detected in a BD Accuri C6 Flow cytometer (BD Biosciences, CA, USA) and the data were analyzed using FlowJo software (BD Biosciences, CA, USA).

### MMP and ROS measurements

Briefly, the treated cells were incubated with JC-1 (Beyotime) for mitochondrial membrane potential (MMP) detection, with MitoSOX (5 μM, Invitrogen, M36008) for mitochondria-derived ROS evaluation. The NPCs were harvested and analyzed by flow cytometry to detect the changes of MMP. The samples stained with MitoSOX and Mitotracker red were imaged with a fluorescence microscope (Olympus Inc., Tokyo, Japan), and the fluorescence intensity was measured by ImageJ software (Bethesda, MD, USA).

### Immunofluorescence

The samples were permeated in 1% Triton X-100 for 10 min, blocked with 1% goat serum for 1 h at room temperature, washed with PBS and incubated with primary antibodies (1:100) in a humid chamber overnight at 4 °C. Next, the slides were washed with PBS and incubated with Alexa Fluor^®^488- or Alexa Fluor^®^594-conjugated secondary antibodies (1:400) in the dark for 1 h at 37 °C, followed by DAPI staining for 5 min. Images were captured under a fluorescence microscope (Olympus Inc., Tokyo, Japan), and analyzed using ImageJ software (Bethesda, MD, USA).

### Real-time PCR

The total RNA of cells was extracted using TRIzol reagent and quantitated by a NanoDrop ND-2000 spectrophotometer (Thermo Scientific, MA, USA). The complementary DNA was synthetized from 1 μg of total RNA with the PrimeScript-RT Reagent Kit (Takara, Japan) and then amplified with the SYBR^®^ Premix Ex Taq™ II kit (Takara, Japan) according to the manufacturer’s protocols. The relative mRNA expressions were calculated using the 2^−ΔΔCt^ method and normalized to the expression of β-actin.

### Lentivirus transfection

The lentivirus for Nrf2 knockdown and Nrf2 overexpression were obtained from GeneChem (Shanghai, China). NPCs at 30% confluency were transfected with lentivirus, following the manufacturer’s protocols. Transfection efficiency was measured by western blotting.

### SA-β-gal staining

The NPC senescence was evaluated by senescence-associated β-galactosidase (SA-β-gal) staining kit (Beyotime) according to the protocols. Briefly, cells implanted into six-well plates were fixed with 0.2% glutaraldehyde for 10 min at room temperature, and subsequently stained with X-gal staining solution overnight at pH 6.0. The aging NPCs were positive for SA-β-gal staining and quantified for statistical analysis.

### TUNEL assay

The NPC apoptosis were detected by the terminal deoxynucleotidyl transferase (TdT) dUTP nick-end labelling (TUNEL) assay. After being fixed in 4% paraformaldehyde for 1 h and incubated with 0.3% Triton X-100 for 5 min, NPCs were stained with the TUNEL Assay Kit (Beyotime) and DAPI. Finally, the slides were randomly selected and captured under a fluorescence microscope (Olympus Inc., Tokyo, Japan) to count TUNEL-positive cells.

### Immunohistochemistry

After fixation in formaldehyde and decalcification, the paraffin-embedded samples were sectioned at 5 μm for immunohistochemical staining. The sections were deparaffinized, rehydrated and treated with 3% hydrogen peroxide for 10 min at room temperature to eliminate endogenous peroxidase activity. Afterwards, the sections were incubated with 0.4% pepsin (Sangon Biotech, Shanghai, China) for 30 min at 37 °C to retrieve the antigen and subsequently blocked with 1% goat serum albumin for 30 min at room temperature. After incubation with primary Nrf2 antibody (1: 200) overnight at 4 °C, the sections were incubated with an appropriate secondary antibody for 1 h at 37 °C, and counterstained with hematoxylin. Images were captured under a light microscope (Olympus Inc., Tokyo, Japan).

### Puncture-induced IDD model

The SD rats (n = 21) were obtained from the Experimental Animal Center of Third Military Medical University. These rats were randomly divided into three groups (the control group, IDD group and IDD+Kin group, n = 7 per group) and anesthetized with an intraperitoneal injection of 2% (w/v) pentobarbital (50 mg/kg) before surgery. After palpating on the coccygeal vertebrae, we exposed the Co7/8 disc through a small sagittal skin incision under aseptic conditions and subsequently punctured it with a needle (27G) as previously described [45]. The depth of puncture was 4 mm. The needle was rotated 360° and kept in the disc for 30 s. Then the surgical incision was sutured. The rats in IDD+Kin group were intraperitoneally injected with Kinsenoside (10 mg/kg per body weight) every 3 days [19], while other rats were injected with same amount of PBS every 3 days until they were sacrificed.

### Magnetic resonance imaging (MRI) examination

Four weeks after surgery, the signal and structural changes of discs were evaluated using sagittal T2-weighted images collected with a 7.0 T animal magnet (Bruker Pharmascan, Germany). The settings for obtaining sagittal T2-weighted images were based upon the parameters described in a previous study [46]. Images were assessed in a double-blind manner according to the Pfirrmann grading system [47].

### Histological analysis

The rat disc samples were fixed in 4% paraformaldehyde for 48 h and decalcified in 10% EDTA for 1 month. Then, the samples were dehydrated, embedded in paraffin and sectioned at 5 μm. The sections of each disc were stained with Safranin O/fast green and evaluated by the histological score based on the morphology of discs [45].

### Statistical analysis

All data are expressed as the mean ± SD of at least three independent experiments. Statistical analysis was performed with GraphPad Prism 7.0 (GraphPad Software Inc., CA, USA) using one-way analysis of variance (ANOVA) followed by Tukey’s test for assessing differences between groups. The Kruskal–Wallis H test was used for analyzing nonparametric data (Pfirrmann grades). The differences between groups were considered statistically significant when *p* < 0.05.

## Supplementary Material

Supplementary Figure
