# Supplementary material for: Kinsenoside ameliorates intervertebral disc degeneration through the activation of AKT-ERK1/2-Nrf2 signaling pathway
Source: Aging (Albany NY). 2019 Sep 23;11(18):7961–77. doi: 10.18632/aging.102302 (PMC6781981; doi:10.18632/aging.102302)
Supplement: Supplementary Figure [file aging-11-102302-s001.pdf]

SUPPLEMENTARY FIGURE

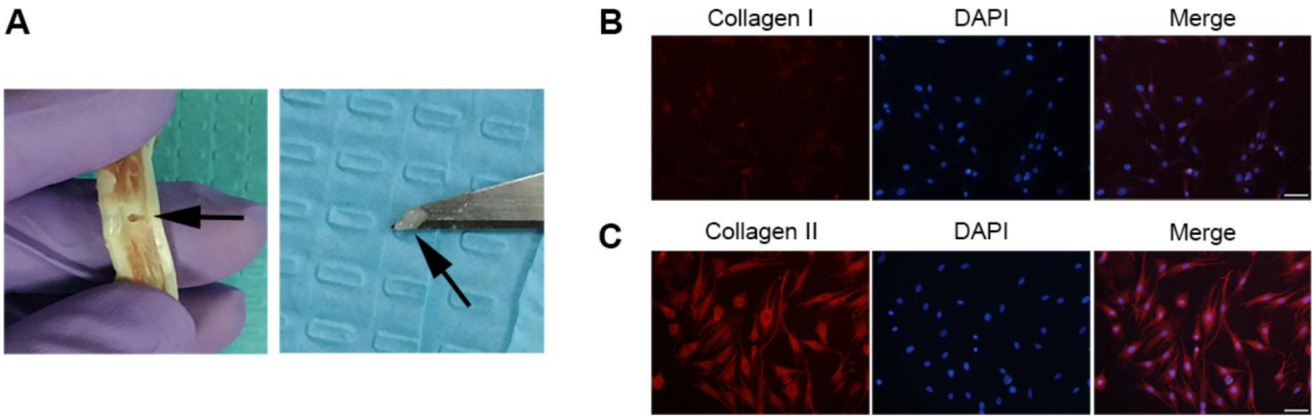

Supplementary Figure 1. Extraction steps and identification methods of NPCs. (**A**) The gel-like NP tissues were separated from the tails of rats using a pointed blade. (**B**, **C**) Immunofluorescence results of Collagen I and Collagen II in NPCs (scale bar: 50μm).
